# Physiologic expansion of human heart-derived cells enhances therapeutic repair of injured myocardium

**DOI:** 10.1186/s13287-019-1418-3

**Published:** 2019-11-04

**Authors:** Seth Mount, Pushpinder Kanda, Sandrine Parent, Saad Khan, Connor Michie, Liliana Davila, Vincent Chan, Ross A. Davies, Haissam Haddad, David Courtman, Duncan J. Stewart, Darryl R. Davis

**Affiliations:** 1 University of Ottawa Heart Institute, Division of Cardiology, Department of Medicine, University of Ottawa, H3214 40 Ruskin Ave, Ottawa, ON K1Y4W7 Canada; 2 Ottawa Hospital Research Institute, Division of Regenerative Medicine, Department of Medicine, University of Ottawa, Ottawa, K1H8L6 Canada; 3 University of Ottawa Heart Institute, Division of Cardiac Surgery, Department of Medicine, University of Ottawa, Ottawa, K1Y4W7 Canada; 4 University of Saskatchewan, Saskatoon, S7N 0W8 Canada; 5 Department of Cellular and Molecular Medicine, University of Ottawa, Ottawa, K1H8M5 Canada; 6 Regenerative Medicine Program, Ottawa Hospital Research Institute, Ottawa, K1H8L6 Canada

**Keywords:** Heart failure, Cell manufacturing, Cell therapy, Adult progenitor cells

## Abstract

**Background:**

Serum-free xenogen-free defined media and continuous controlled physiological cell culture conditions have been developed for stem cell therapeutics, but the effect of these conditions on the relative potency of the cell product is unknown. As such, we conducted a head-to-head comparison of cell culture conditions on human heart explant-derived cells using established in vitro measures of cell potency and in vivo functional repair.

**Methods:**

Heart explant-derived cells cultured from human atrial or ventricular biopsies within a serum-free xenogen-free media and a continuous physiological culture environment were compared to cells cultured under traditional (high serum) cell culture conditions in a standard clean room facility.

**Results:**

Transitioning from traditional high serum cell culture conditions to serum-free xenogen-free conditions had no effect on cell culture yields but provided a smaller, more homogenous, cell product with only minor antigenic changes. Culture within continuous physiologic conditions markedly boosted cell proliferation while increasing the expression of stem cell-related antigens and ability of cells to stimulate angiogenesis. Intramyocardial injection of physiologic cultured cells into immunodeficient mice 1 week after coronary ligation translated into improved cardiac function and reduced scar burden which was attributable to increased production of pro-healing cytokines, extracellular vesicles, and microRNAs.

**Conclusions:**

Continuous physiological cell culture increased cell growth, paracrine output, and treatment outcomes to provide the greatest functional benefit after experimental myocardial infarction.

## Background

Mechanical and pharmaceutical advances in cardiac care have dramatically reduced the mortality associated with myocardial infarction. As a result, health care systems are faced with a growing number of patients living with chronic heart failure—a diagnosis that still carries a 5-year mortality approaching 50% [1, 2]. This observation reflects the ability of current therapies to slow the progression of heart failure without addressing the loss of functional myocardium.

Over the past 20 years, a number of cell therapies have been tested as cardiac therapeutics with inconsistent or, at best, modest efficacy. Clinical studies using autologous heart-derived cell products have been much more encouraging with all trials universally demonstrating safety and promising hints of efficacy [3–7]. In comparison to other heart-derived cell products, pre-clinical studies from 45+ independent labs have shown that heart explant-derived cells (EDCs) or their expanded progeny (cardiosphere-derived cells) contain a complementary admixture of progenitor cells that restore myocardial function largely through indirect paracrine stimulation of endogenous repair [8]. Although EDCs are considerably more apt to grow into working heart tissue than cardiosphere-derived cells, clinical delivery has not been possible as the human cell “dose,” estimated to be 25–75 M based on cardiosphere-derived cell trials, greatly exceeds production capacity using traditional methods [9]. If a realistic dose of EDCs can be attained, EDCs may outperform other heart-derived cells in the clinic while shortening time in ex vivo cell culture to reduce costs and limit the prospects for malignant or senescent transition. As such, we focused on cell production, an important and often understudied area of research, to identify novel means of enhancing EDC cell culture outcomes.

We initially focused on replacing the standard lab grade materials with fully defined and sourced factors. Translating promising pre-clinical cell products to the clinic always promises to be difficult as standard cell culture media is typically supplemented with ill-defined or xenobiotic components, such as fetal bovine serum. Concerns arise that these “unknown” components increase the risk of non-human pathogen contamination, induce unwanted immune responses, or have inconsistent effects on cell phenotype [10]. Despite the large body of literature on serum-free media culture for stem cell expansion, uptake has been slow with 80% of all human mesenchymal stem cell regulatory submissions describing the use of fetal bovine serum during manufacturing [11]. This may be attributable to regulatory bodies accepting the use of well-characterized foreign materials in early proof of safety or efficacy trials. But all express a strong preference towards defined xenogen-free materials when a product enters phase 3 or 4 clinical testing. Based on evidence that subtle alterations in the constituents used for cell culture alter regenerative cell performance, “late in the day” reformulation and retesting have the potential to impart considerable costs while ultimately blunting cell efficacy culture [12–14]. Finally, the ethics of collecting fetal blood through fetal cardiac puncture without anesthesia is troublesome and makes the search for other solutions highly desirable [15]. Therefore, we initially explored the hypothesis that serum-free xenogen-free defined media would support EDC cell growth without adversely impacting upon therapeutic repair of injured myocardium using a mouse model of ischemic injury.

Cell phenotype is not determined by media composites or cytokines alone. The oxygen content and temperature within the cell culture environment also play a key role in guiding cell fate. Traditionally, lab quality and most clinical grade cells are cultured in standalone carbon dioxide and temperature-controlled incubators exposed to ambient air (21% oxygen, 5% carbon dioxide), which is much higher than the in situ cardiac microenvironment (5% oxygen). Previous work has shown that maintaining cells within physiological 5% oxygen conditions reduces levels of intracellular reactive oxygen species and cell senescence while increasing genomic stability [16, 17]. During processing, cells are removed from the supportive incubators for interventions or media changes within standalone biosafety cabinets which exposes cells to ambient oxygen (21%) and temperatures (21 °C). Given the impact of the cell culture environment on cell stress responses [18, 19], we tested the hypothesis that ex vivo expansion of human heart-derived cells in constant physiologic conditions (5% oxygen, 37 °C) that mimic the in vivo microenvironment would enhance human cell culture outputs using an established immunodeficient mouse model of ischemic cardiomyopathy.

## Methods

### Cell isolation and culture

EDC cultures were established from atrial appendages or ventricular biopsies obtained from patients undergoing clinically indicated procedures. Inclusion criteria for tissue donors consisted of patients between the ages of 18 and 80 who (1) required cardiac surgery for coronary artery bypass grafting and/or valve surgery, or (2) were undergoing routine post-heart transplant screening for allograph rejection. Exclusion criteria included chronic infectious diseases (such as hepatitis, human immunodeficiency virus), pregnant women, or active sepsis.

**Fig. 1:**
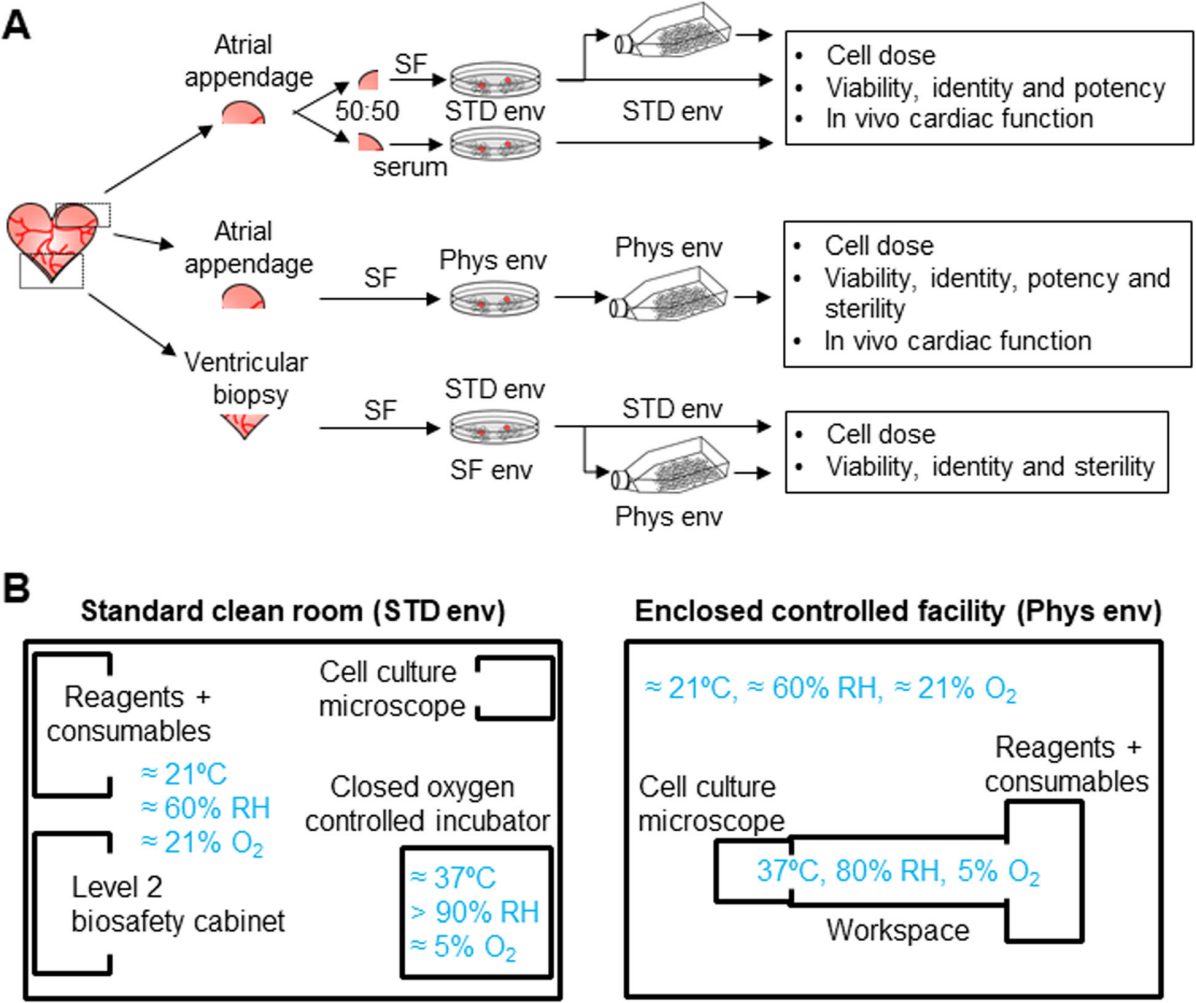
Study outline. **a** Schematic contrasting cell culture within a standard clean room environment (STD env) with an isolated facility that continually maintains normal cardiac physiological conditions (37 °C, 80% relative humidity (rh), and 5% oxygen (O_2_)). **b** Schematic outlining human atrial and ventricular tissue processing compared throughout the study and key outcomes

As specified, tissue samples were cultured within standalone oxygen-controlled incubators in a clean room level 2 biosafety cabinet environment (STD env) or an enclosed, clinical grade, cell manufacturing facility under continuous physiological conditions (Phys env, Fig. 1a). The latter is being performed within the Ottawa Hospital Research Institute Cell Manufacturing Facility using SF GMP compatible standard operating procedures within ISO5 BioSpherix Xvivo Isolators (Biospherix Inc). These interconnected modules provide a continuous atmosphere (5% oxygen, 5% carbon dioxide), relative humidity (80% RH), and temperature (37 °C)-controlled environment to mirror the normal in vivo cardiac microenvironment [17].

As previously described, tissue biopsy samples were minced, washed, and digested with standard collagenase IV (Life Technologies) or a GMP-grade blend of collagenase I/II (Roche) [20] before plating on GMP-grade fibronectin (Roche)-coated plates in standard cardiac explant media (Iscove’s modified Dulbecco’s medium, 20% fetal bovine serum, 100 U/ml penicillin G, 100 μg/ml streptomycin, 2 mmol/l l-glutamine, and 0.1 mmol/l 2-mercaptoethanol; all sourced from Life Technologies) or GMP-grade serum-free xeno-free medium (SF; Nutristem SF- XF-, Biological Industries) at physiological (5%) oxygen tension [9, 21–26]. EDCs that spontaneously emerged from the plated tissue were harvested up to 4 times every 7 days using TrypLE Select (Life Technologies) with enumeration using a Neubauer hemocytometer. The effects of static expansion on cell numbers and phenotype were investigated using aliquots of harvested cells seeded at 10% confluency on fibronectin-coated cultureware for 7 days. Given that EDC culture provides a constant output return proportional to the amount of tissue plated and the need for larger cell numbers, right atrial appendage specimens were used for all experiments in this proof of concept study (Fig. 1b). Ventricular tissue was used in cell proliferation experiments to evaluate the ability of GMP SF culture conditions to support cell growth from a tissue source readily harvested for clinical application (Fig. 1b).

Circulating angiogenic cells (CACs) were isolated from blood samples donated by patients undergoing clinically indicated procedures [26]. Mononuclear cells were isolated using density-gradient centrifugation (Histopaque 1077; Sigma-Aldrich) and placed in culture for 4–6 days in endothelial basal media (EBM-2; Clonetics) supplemented with EGM-2-MV-SingleQuots (Clonetics). CACs were harvested by mechanical dissociation for experimentation within 7 days of starting culture. Commercially sourced human umbilical vein endothelial cells (HUVECs) were cultured according to the manufacturer’s directions (Lonza).

### Antigenic profiling

The effects of SF GMP conditions on the phenotypic signature of EDCs were investigated using a custom flow cytometry panel to evaluate expression of cardiac, endothelial, hematopoietic, mesenchymal, and stem cell identity. Flow cytometry (Guava easyCyte 8HT flow cytometer; Millipore) was performed using monoclonal antibodies and similarly conjugated isotype-matched controls for ATP-binding cassette sub-family G member 2 (abcg2; FAB995P, R&D Systems), alpha smooth muscle actin (α-SMA; ab66133, Abcam), Cadherin 11 (FAB17901G, R&D Systems), CD29 (FAB17781P, R&D Systems), CD31 (FAB3567F, R&D Systems), CD44 (FAB4948P, R&D Systems), CD51 (FAB3050A, R&D Systems), CD79 (FMC020, R&D Systems), CD73 (FMC020, R&D Systems), CD90 (FMC020, R&D Systems), CD105 (FMC020, R&D Systems), CD133 (130-090-826, Miltenyi Biotec), CD146 (FAB932F, R&D Systems), CD166 (FAB6561P, R&D Systems), c-Kit (9816-11, Southern Biotech), discoidin domain receptor 2 (DDR2; ab63337, Abcam), Nestin (IC1259F, R&D Systems), platelet-derived growth factor receptor alpha (PDGFR; FAB1264A, R&D Systems), stage-specific embryonic antigen-1 (SSEA-1; FAB2155A, R&D Systems), and a cocktail of hematological markers that included CD11b, CD34, CD45, and CD79A (FMC020, R&D Systems) (Additional file 1: Table S1). A minimum of 20,000 events were detected after fluorescent compensation using unlabeled controls. Positive cells were defined as the percentage of the population falling above the 99th percentile of the relevant isotype control (FlowJo v. 10, TreeStar Inc.).

### Conditioned media for angiogenesis, CAC migration, and paracrine profiling

Conditioned media was obtained from EDCs after 48 h of culture in 1% oxygen 1% serum conditions. The paracrine signature of EDCs was profiled using an unbiased protein array (RayBio cytokine antibody series G or R&D Proteome Profiler Human XL cytokine array ARY022B). Nanoparticles were isolated using ExoQuick-TC Exosome Precipitation Solution (System Biosciences) for nanoparticle tracking analysis (NanoSight LM10; Malvern Instruments) and microRNA (miRNA) profiling (NanoString Technologies). The capacity of EDCs to promote angiogenesis was assessed using a growth factor-depleted matrigel assay (ECM625, Millipore). HUVECs were seeded on matrigel with stem cell conditioned media or serum-free Dulbecco’s modified Eagle’s medium supplemented with 100 mM VEGF (positive media control). After 18 h of incubation, each well was imaged and reconstructed using imaging software to allow for measures of cumulative tubular growth (NeuronJ; National Institutes of Health) [25, 26]. Stem cell recruitment was assessed by plating 4000 human CACs suspended in serum-free Dulbecco’s modified Eagle’s medium into the upper well of a fibronectin-coated transwell plate (24 wells, 3.0 mm pores, Corning) with EDC conditioned media placed in the bottom well. Serum-free Dulbecco’s modified Eagle’s medium containing 100 ng vascular endothelial growth factor (VEGF) was used as an unbiased control to normalize individual variations in CAC migration. After 24 h of normoxic incubation, CACs that had successfully migrated through the polycarbonate membrane were fixed (4% para-formaldehyde) and nuclei were stained with 4′,6-diamidino-2-phenylindole (DAPI; Sigma Aldrich). Fluorescent microscopy (× 10 magnification, 6 random fields) was used to determine the average number of cells per random field (Image J, ICTN plug-in, National Institutes of Health) [25, 26].

### Cardiogenic differentiation

The effect of variable culture conditions on the ability of EDCs to adopt a cardiac phenotype was assessed by plating 20,000 cells/cm^2^ within cardiogenic media [9, 21, 26]. Cardiogenic media consisted of low glucose Dulbecco’s modified Eagle’s media, MCDB-201 media, dimethylsulfoxide, l-ascorbic acid, 0.01% insulin-transferrin-selenium liquid media supplement, linoleic acid-albumin, penicillin-streptomyin, dexamethasone, 2-mercaptoethanol, recombinant human fibroblast growth factor 8b, recombinant human fibroblast growth factor 4, recombinant human protein Dickkopf-related protein 1, and recombinant human bone morphogenetic protein 2 (all from Life Technologies) [27]. After 7 days of culture, cells were harvested for flow cytometry (alpha smooth muscle actin (α-SMA; ab125266, Abcam), cardiac troponin T (cTnT; ab66133, Abcam), or von Willebrand factor (vWF; 11778-1-AP, ProteinTech)).

### In vivo testing

Experimental myocardial infarctions were performed in 39 male non-obese diabetic severe combined immunodeficient (NOD-SCID) mice by permanent ligation of the left coronary (LC) artery [21–23, 25, 26]. Animals were injected with buprenorphine (0.05 mg/kg; subcutaneous) 1 h prior to surgery and twice daily thereafter for 3 days. During the ligation, mice were intubated, anesthetized using isoflurane, and maintained at physiologic temperatures. Upon closure, animals were injected with 0.5 ml of saline (subcutaneous). Seven days after ligation, 100,000 EDCs were injected into the myocardium along the infarct border and at the cardiac apex using transthoracic echocardiographic guidance (VisualSonics). Five mice died prior to completion of the study and were excluded from analysis (*n* = 3 serum EDC-treated mice and *n* = 2 SF EDC-treated mice). Left ventricular ejection fraction was evaluated 21 and 28 days after left coronary artery (LCA) ligation to assess the functional effects of each cell therapy. After the last assessment of myocardial function, the mice were euthanized and hearts excised for histology or quantitative polymerase chain reaction (qPCR) analysis. Myocardial retention of transplanted cells was assessed in a subset of mice using qPCR for non-coding human alu repeats [26]. Left ventricular genomic DNA was extracted, and qPCR was performed with transcript-specific hydrolysis primer probes. The remaining hearts were fixed with 4% paraformaldehyde, embedded in optimal cutting temperature compound, and sectioned. Tissue viability within the infarct zone was calculated from Masson’s trichrome (Life Technologies) stained sections by tracing the infarct borders manually and using ImageJ software to calculate the percent of viable myocardium within the overall infarcted area [21–23, 25, 26]. EDC engraftment was confirmed by staining sections for human nuclear antigen (HNA; SAB4500768, Sigma-Aldrich) while EDC fate was established by staining sections for co-segregation with α-SMA, cTnT, and vWF. Contributions of EDC therapy to capillary density were assessed by staining sections for isolectin B4 (B-1205, Vector Laboratories). All functional evaluations were conducted and analyzed by investigators blinded to the animal’s treatment group.

### EDC stability and delivery testing

A French 11Fr (3.7 mm) TREK Coronary Dilation Catheter (Abbott Vascular) and a French 8Fr (2.7 mm) NOGA MyoStar Intramyocardial Injection Catheter (Biosense Webster) were used to assess the impact of catheter delivery on cell viability. After coating the internal channel of both catheters with 25% human albumin (A2153, Sigma), harvested EDCs were suspended in Plasmalyte A (2B2544X, Baxter) with 2.5% human albumin for catheter delivery. After injecting 2 million cells through the internal channels, viability testing (Trypan Blue) and delivery counts were performed. The long-term stability of EDCs for transport between institutions was established using EDCs drawn up into BD Luer-Lok syringes and stored at 4 °C for 18 h. Cell viability using Trypan Blue (H7901, Sigma) exclusion was determined at time 0 (prior to loading the syringes) and after 18 h.

### Statistical analysis

Data are expressed as mean ± standard error of the mean. To determine if differences existed within groups, data was initially analyzed by a one-way ANOVA. If such differences existed, Bonferroni’s corrected *t* test was used to determine the group(s) with the difference(s) (Prism 6.01, GraphPad). Differences in categorical measures were analyzed using a chi-square test. A final value of *p* ≤ 0.05 was considered significant for all analyses.

## Results

### Serum-free culture conditions support cell growth from atrial and ventricular tissue

As shown in Table 1, atrial appendages or ventricular biopsies were harvested from patients undergoing clinically indicated cardiac surgery or post-transplant rejection surveillance, respectively. Primary EDC cultures were established by plating half of each atrial appendage specimen in standard serum-supplemented media and half in SF medium (50:50 split by mass, Fig. 1a) within standalone oxygen-controlled incubators and level 2 biosafety cabinets in a standard clean room facility (STD env, Fig. 1b). To ensure that all the components used within explant culture conformed to GMP standards, the effect of transitioning explant culture from commercial grade collagenase IV to GMP compliant collagenase I/II was first evaluated. In contrast to previous work demonstrating the effect of divergent culture practices on EDC biology [12–14], GMP grade collagenase did not influence either overall cell culture yields (20 ± 5 versus 21 ± 6 × 10^6^ cells per mg tissue plated, respectively; *p* = 0.92) or the major sub-population content at each serial harvest from the plated tissue (Additional file 1: Figure S1).

**Table 1:**
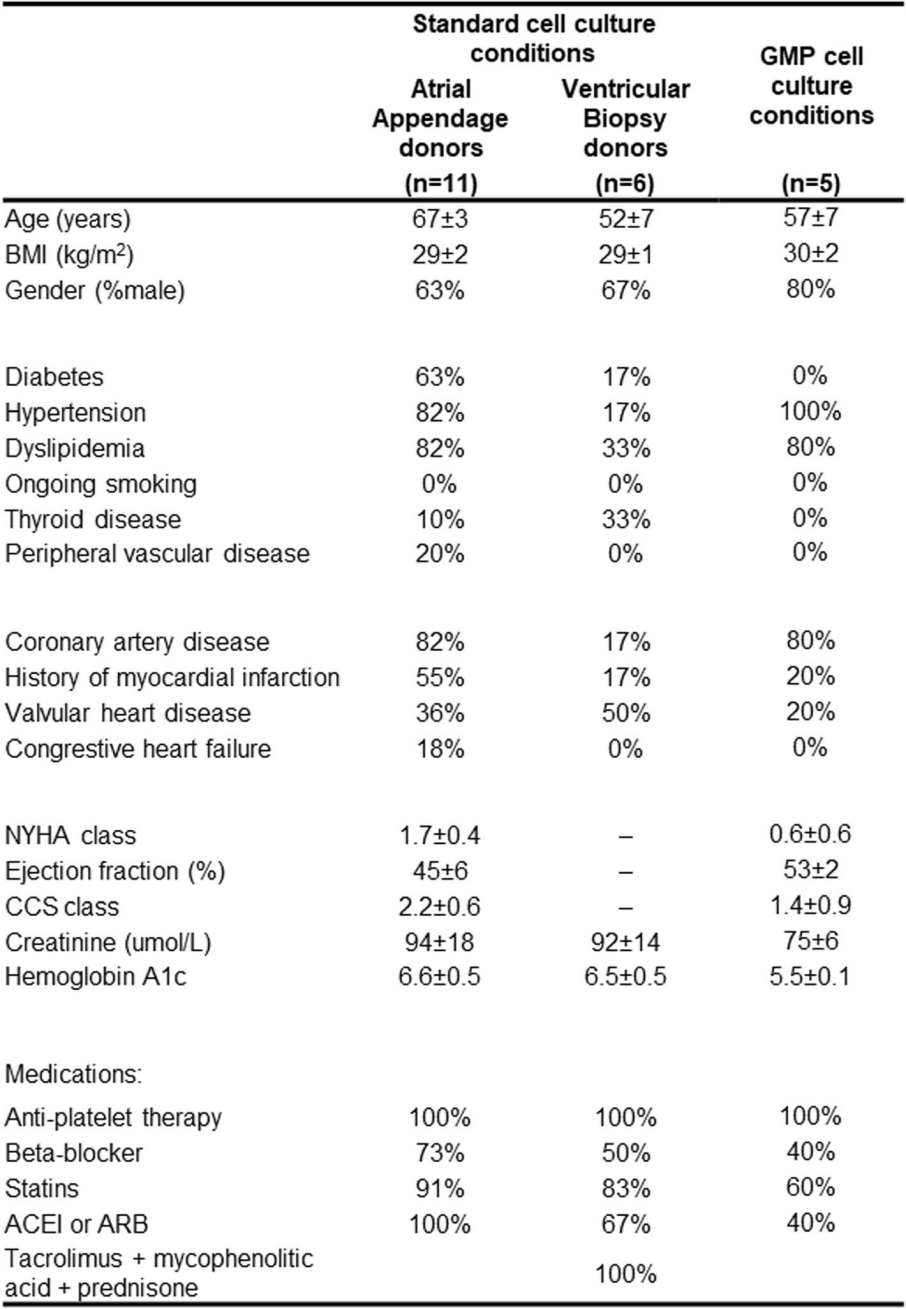
Patient clinical characteristics

*ACEI* angiotensin-converting enzyme inhibitors, *ARB* angiotensin II receptor blockers, *BMI* body mass index, *CCS* Canadian Cardiovascular Society, *NYHA* New York Heart Association

**Fig. 2:**
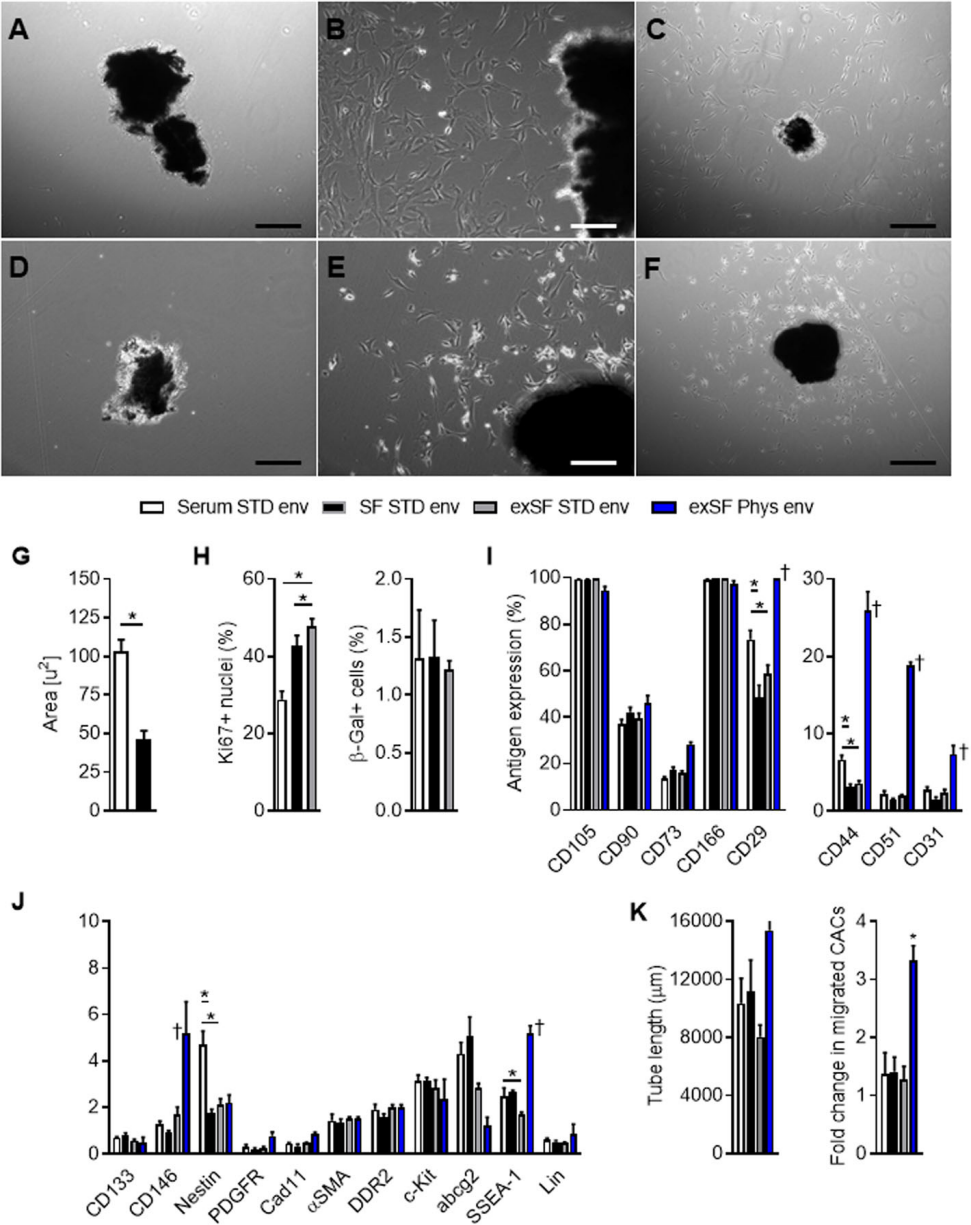
Effects of serum-free good manufacturing practices (GMP) compatible culture conditions on explant-derived cell (EDC) phenotype. Representative brightfield images of plated cardiac tissue fragments and EDC outgrowth under 20% serum conditions. **a** 1 day post-plating. **b** 3 days post-plating. **c** 7 days post-plating. Representative brightfield images of plated cardiac tissue fragments and EDC outgrowth under serum-free conditions. **d** 1 day post-plating. **e** 3 days post-plating. **f** 7 days post-plating. **g** Flow cytometry demonstrating that cells cultured in SF STD env conditions were smaller and more homogenous than cells cultured in serum STD env conditions. **h** Immunohistochemical staining for the cell cycle-associated protein Ki67 in conjunction with DAPI (left panel). Senescence-associated beta-galactosidase+ (β-Gal+) EDCs identified under phase-contrast microscopy by the presence of intracellular hydrolyzed X-galactosidase (right panel). **i**, **j** Flow cytometry analysis of phenotypic composition of EDCs. **k** Effect of cell culture conditions on the ability of EDCs to stimulate human umbilical vein endothelial cells (HUVECs) tubule formation (left panel) or attract circulating angiogenic cells (CACs) across a transwell membrane (right panel; expressed as fold change number of migrated cells compared to basal media containing 100 ng vascular endothelial growth hormone (VEGF; normalization control)). *p ≤ 0.05, **p ≤ 0.01, *n* = 4 to 5 cell cultures per group. abcg2, ATP-binding cassette sub-family G member 2; cad11, Cadherin-11; DDR2, discoidin domain receptor tyrosine kinase 2; Lin, hematological lineage cocktail; PDGFR, platelet-derived growth factor receptor; SSEA-1, stage-specific embryonic antigen-1

When tissue biopsies were cultured using SF xenogen-free media, brightfield images demonstrated that the EDCs which spontaneously emerged from tissue were smaller and more uniform in size (Fig. 2a–f). This impression was confirmed through flow analysis of the forward (a correlate of cell surface area or size) and side (a correlate of granularity or internal complexity) scatter within harvested cells (Fig. 2g). Overall, SF EDCs demonstrated a lower forward scatter and reduced elliptical area of 95% containment (46 ± 6 versus 103 ± 7 square units for cells cultured in standard media, arbitrary units; *p* = 0.002).

Given the commonly encountered issues surrounding proliferation and senescence when transitioning cells to SF media, the influence of these parameters on culture outcomes was evaluated. Despite having little effect on overall cell culture yields from plated biopsies (19 ± 3 versus 22 ± 4 × 10^6^ cells per mg tissue plated, *p* = 0.57 versus serum-based media), SF media conditions increased the number of Ki67+ cells in culture (*p* = 0.008 versus serum EDCs) with no effect on cell senescence (Fig. 2h).

The effects of SF conditions on the antigenic identity of EDCs were profiled using a custom panel designed to identify cells expressing differentiated (α-SMA, DDR2, lin), endothelial (CD31, CD44, CD51), mesenchymal (CD29, CD73, CD90, CD105, CD146, CD166), and stem cell (abcg2, cad11, CD133, c-Kit, nestin, PDGFR, SSEA-1) markers within EDCs (Additional file 1: Table S1). As shown in Fig. 2i, j, SF culture conditions had only minor effects on the CD29+ (Δ-25 ± 5%, *p* = 0.05), CD31+ (Δ-1.2 ± 0.3%, *p* = 0.05), CD44+ (Δ-3.5 ± 0.3%, *p* = 0.001), and Nestin+ (Δ-3.0 ± 0.2%, *p* = 0.04) content within EDCs.

The effects of SF conditions on the regenerative potency of EDCs were investigated using established in vitro measures of cell potency [21–23]. As shown in Fig. 2k, application of conditioned media to HUVECs within a cytokine-depleted matrigel angiogenesis assay or CACs across a transwell migration assay demonstrated that altering EDC culture conditions had little effect on angiogenesis or CAC recruitment. Similarly, exposure of EDCs to cell conditions that stimulate the adoption of cardiac markers demonstrated that SF culture had no effect on the ability of cells to adopt a cardiomyocyte (cTnT+, *p* = 0.55 versus serum STD env) or endothelial (vWF+, *p* = 0.22 versus serum STD env) lineage (Additional file 1: Figure S2). Tissue source did not alter EDC proliferation within SF GMP conditions as culture yields from plated ventricular biopsies were maintained (1.7 ± 0.3 versus 1.2 ± 0.5 million cells cultured per biopsy sample; *p* = 0.45 versus serum STD env). Taken together, these data suggests that SF xenogen-free culture conditions support the ex vivo growth of EDCs from multiple tissue sources with only minor effects on the phenotypic signature of cells but, in contrast to alternative changes in media formulation that negatively impact cell function [12, 13], no signal to suggest an inferior regenerative cell product.

### Serum-free compatible culture conditions support EDC expansion

Given that initial EDC culture yields are limited by a constant return proportional to amount of tissue plated [9], the influence of straightforward sub-culture within adherent cultures was investigated as a means of attaining clinically meaningful cell “doses” (exSF groups). Plating SF EDCs within SF media in a standard clean room environment provided a 5.5 ± 1.1-fold increase in cell numbers over 7 days (population doubling time, 73 ± 11 h). As shown in Fig. 2h, SF expansion did not increase senescence while maintaining robust numbers of Ki67+ cells (*p* = 0.18 versus SF STD env). Excluding a slight decrease seen in the proportion of SSEA-1+ cells (Δ1.0 ± 0.1%, *p* = 0.01 vs SF culture), SF expansion did not alter the antigenic profile of SF EDCs (Fig. 2i, j). Reassuringly, as shown in Fig. 2k and Additional file 1: Figure S1, SF expansion did not alter the ability of EDCs to stimulate angiogenesis, recruit CACs, or express cardiac markers. Thus, EDC expansion within adherent SF culture conditions maintains cell phenotype with no significant effects on in vitro measures of regenerative potency.

### Physiologic serum-free cell culture boosts proliferation and potency

**Fig. 3:**
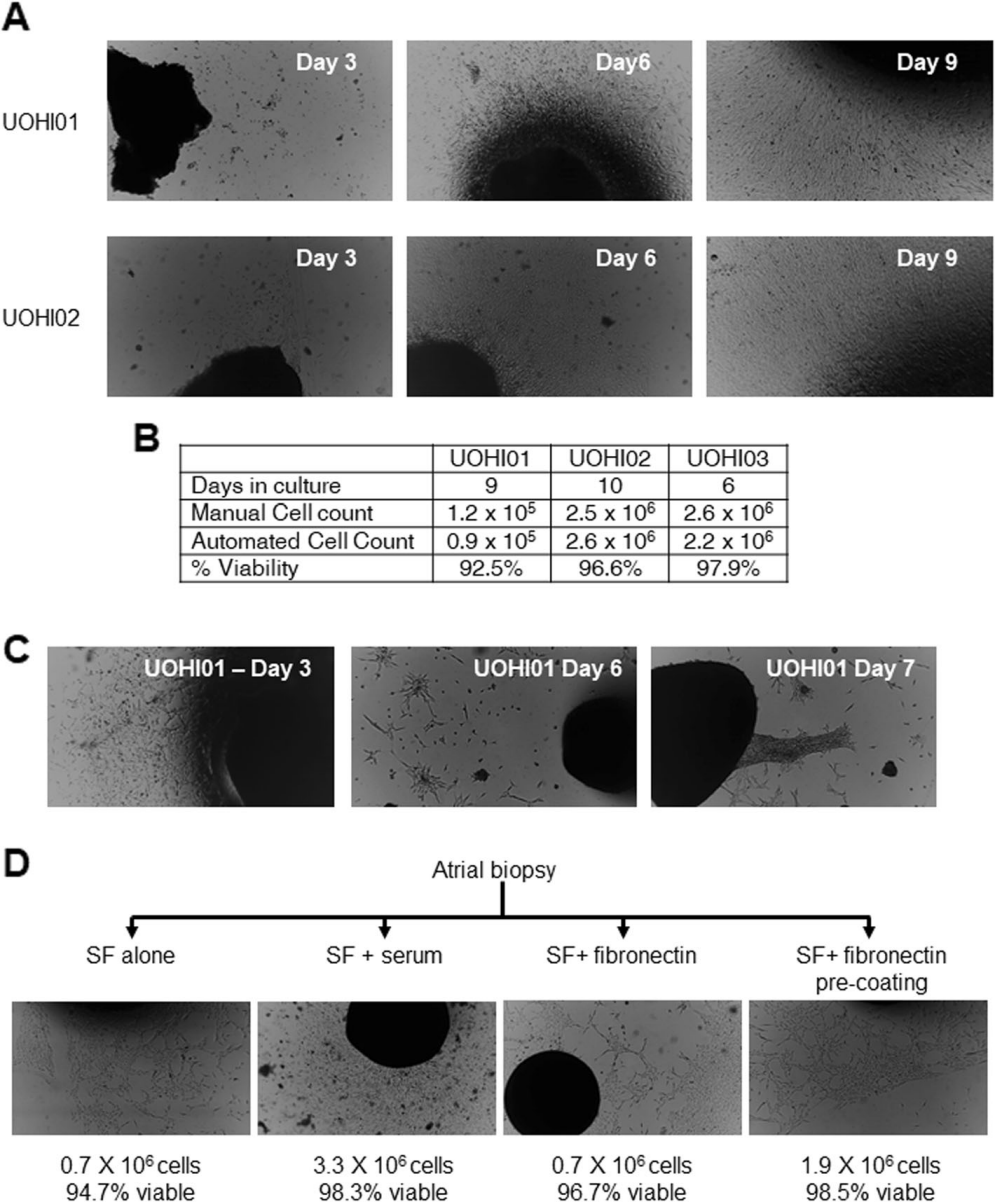
Influence of physiological environment cell culture conditions on explant-derived cell (EDC) culture. **a** Representative images of plated tissue from two initial cell cultures (UOHI01 and UOHI02) demonstrating progressive spontaneous migration of EDCs from the plated tissue to cover the cultureware over 9 days in culture. **b** Table outlining the cell yields and viability of the initial three cell cultures demonstrating the impact of reducing the interval between cell culture harvests. **c** Representative images from the initial cell cultures attempted showing sparse EDC outgrowth after the initial enzymatic harvest and the formation of adherent monolayers that occurred over time. **d** Experimental schemata, representative images, and experimental outcomes demonstrating the influence of altering cell culture conditions on EDC yields after the first enzymatic digestion. SF, serum free


The effect of continuous physiological conditions (80% RH, 5% oxygen, 37 °C) on heart biopsy samples was evaluated using enclosed cell culture conditions within a clinical grade cell manufacturing facility (Phys env). As shown in Fig. 1a, once tissue entered the cell culture modules, all processing, cell culture, and sampling occurred under continuous physiological conditions until the final cell product emerged for experimentation. Using a detailed standard operating procedure reflecting established clean room protocols, a series of atrial appendages underwent testing to confirm protocol feasibility. As shown in Table 1, the clinical features of tissue donors reflected those used in previous clean room trials and previous cell therapy publications [21, 22, 26, 28–30]. Initial growth from plated tissue samples in Phys env was robust (Fig. 3a), and after 9 to 10 days in culture, each sample yielded a predictable number of cells following the first enzymatic collection reminiscent of initial harvests seen during culture within the STD env (Fig. 3b). However, subsequent collections were severely reduced as cells coalesced to form aggregates or failed to emerge around the plated tissue (Fig. 3b). Time-lapse imaging revealed that while cells continued to emerge from the plated tissue after the first enzymatic collection, they appeared to lose purchase on the cultureware and re-enter the tissue (Additional file 2: Movie S1). This observation suggested that brief initial enzymatic digestion (≈ 7.5 min) needed to harvest adherent cells also stripped away fibronectin coating from the plate surface. To test the influence of “re-coating” adherent surfaces after enzymatic harvest, a single atrial appendage was split between culture conditions containing the following: (1) SF media alone, (2) SF media with serum media for 2 days after cell collection, (3) SF media supplement with fibronectin (10 μg/ml), or (4) re-coating cultureware with fibronectin in saline (10 μg/ml) for 30 min after cell collection EDC collection. As shown in Fig. 3c, re-application of fibronectin ensured robust cell numbers during the second cell harvest. Time-lapse profiling revealed that as cells emerged to cover the cultureware, tractional forces are applied to the plated human tissue biopsy by migrating cells (Additional file 3: Movie S2).

**Additional file 2: Movie S1.** Time-lapse imaging of EDCs emerging from plated tissue after the first enzymatic collection following 1 week of culture in Phys Env without fibronectin re-coating.



**Additional file 3: Movie S2.** Time-lapse imaging showing EDCs placing traction on the plated tissue.


Once optimized, the impact of a continuous physiological environment on cell culture outcomes became evident as physiologic conditions prompted heart tissue cultures to attain confluence sooner (≈ 2–5 vs. 7 days) thus shortening the interval between enzymatic collections. Plating harvested cells for static expansion was also accelerated as population doubling times decreased from 73 ± 11 to 29 ± 2 h (*p* < 0.05) with no detectable evidence for contamination (conditioned media culture + endotoxin + gram stain) or attenuated viability. Flow cytometry antigenic profiling demonstrated physiologic culture altered cell marker expression such that the universal mesenchymal stem cell marker CD29 was expressed by all cells while enriching endothelial (CD31, CD44, and CD51) and stem cell (Nestin, SSEA-1) markers (Fig. 2i, j). Interestingly, these antigenic changes were associated with markedly greater stimulation of vascular network formation (≈ 1.5-fold greater, Fig. 2k) and circulating angiogenic cell recruitment (≈ 2-fold greater, Fig. 2k). Taken together, this data indicates SF culture within physiologic environments would enable a cell manufacturing facility to attain intracoronary (≈ 25 M) or intramyocardial (≈ 100 M) doses of a pro-angiogenic cell product within 14 or 24 days, respectively.

### Physiological serum-free cell culture enhances cell-mediated repair of ischemic myocardium

**Fig. 4:**
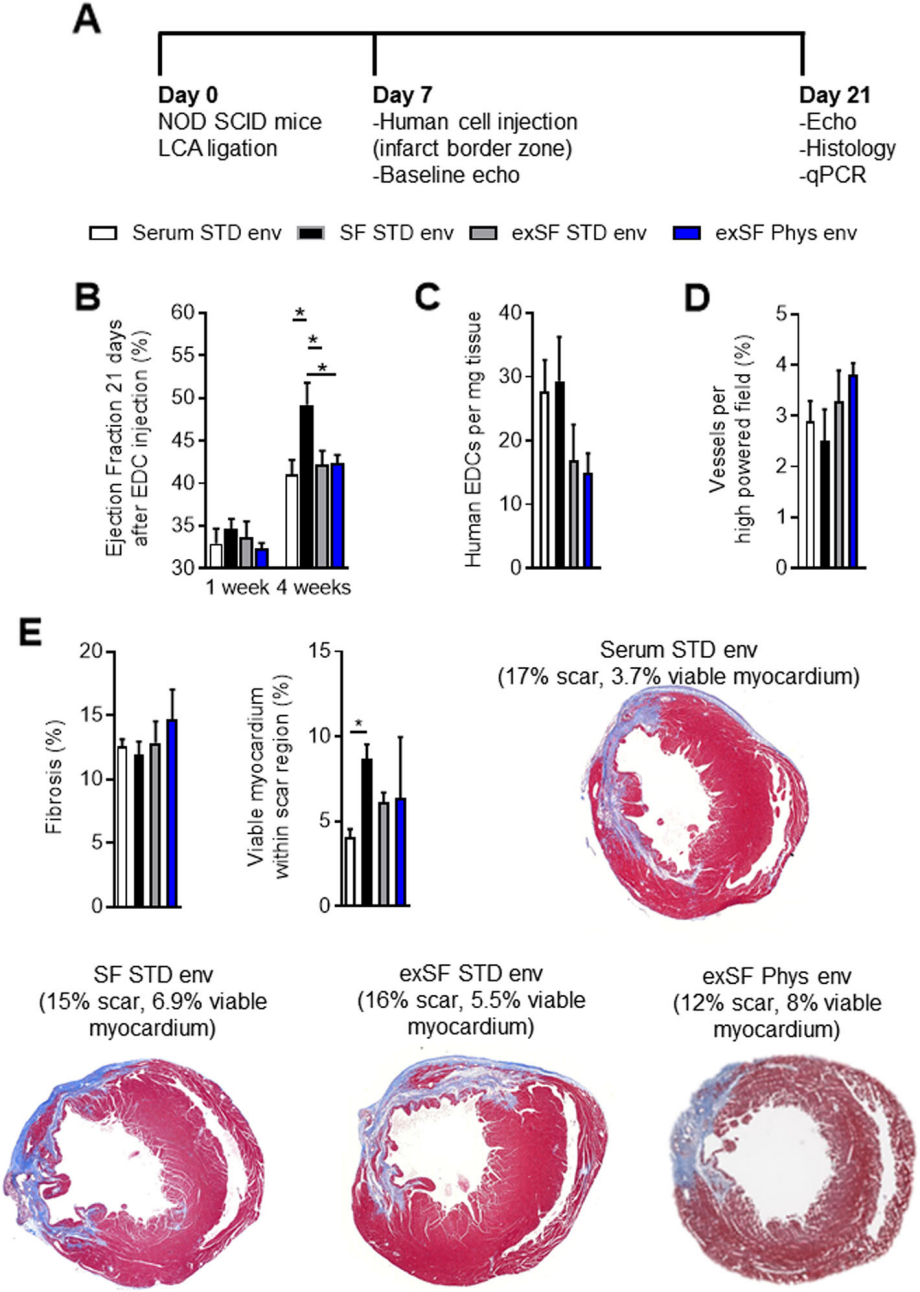
Influence of culture conditions on heart explant-derived cell (EDC) mediated repair of injured myocardium. **a** Schemata of the experimental protocol demonstrating echocardiographic-guided intramyocardial injection of 100,000 EDCs into immunodeficient non-obese diabetic severed combined immunodeficient mice 1 week after left coronary artery (LCA) ligation. EDCs cultured using standard serum media in standard cell culture conditions (Serum STD env), serum-free media in STD cell culture conditions (SF STD env), serum-free media-derived EDCs expanded for 1 week in STD cell culture conditions (exSF STD env), and SF media expanded EDCs cultured entirely within continuous physiologic controlled (exSF Phys Env) culture conditions were compared. **b** Effects of EDC transplant on echocardiographic ejection fraction 3 weeks after cell injection. *n* = 10 to 13 per group. **c** EDC engraftment 3 weeks after intramyocardial injection as determined using quantitative polymerase chain reaction analysis for retained human alu sequences (left panel; *n* = 6 per group) or random field counts of human nuclear antigen+ (HNA+)/4′,6-diamidino-2-phenylindole+ (DAPI+) cells (right panel; *n* = 8 per group). **d** Effects of EDC transplant on vessel density (isolectin B4+) within the peri-infarct region. *n* = 5 per group. **e** Effects of EDCs on the overall percentage infarcted myocardium (left panel; *n* = 5 per group) and percentage of viable myocardium within the infarct zone (right panel; *n* = 6 per group) as determined from Masson’s trichrome staining. *p ≤ 0.05. LCA, left coronary artery; NOD-SCID, non-obese diabetic severe combined immunodeficient; qPCR, quantitative polymerase chain reaction

The influence of SF physiologic cell culture on therapeutic cardiac repair was investigated in a series of immunodeficient mice randomized to echocardiographic-guided injection of serum, SF, or expanded SF EDCs 1 week after LCA ligation (Fig. 4a). As shown in Fig. 4b, all animals had equivalent ejection fractions 1 week after LCA ligation. Animals treated with SF EDCs showed superior improvements in echocardiographic ejection fraction 3 weeks after cell treatment compared to animals receiving traditional serum cultured EDCs (48 ± 3 versus 40 ± 2%, respectively; *p* = 0.046). The regenerative advantages conferred by administering cells cultured in SF conditions were reduced in animals that received equivalent “cell doses” of exSF STD env (41 ± 2%; *p* ≤ 0.05 versus SF EDCs) or Phys Env (42 ± 1%; *p* ≤ 0.05 versus SF EDCs) EDCs to an extent comparable to animals who received cells cultured in standard serum conditions. Unsurprisingly, treatment with SF cultured EDCs had no detectable influence on the modest long-term engraftment of transplanted cells (Fig. 4c). Despite clear improvements in myocardial function, administration of SF STD env or Phys env cultured EDCs had no effect on capillary densities (Fig. 4d and Additional file 1: Figure S3) or the final left ventricular scar burden (Fig. 4e). This lack of effect was likely attributable to increases in viable myocardium seen in SF EDC-treated animals within the infarct itself (Fig. 4e), hinting that SF cultured EDCs promoted myogenesis or myocardial salvage while not reducing the overall scar burden. Thus, SF cultured cells provide an enhanced cell product while SF expansion within STD or Phys Env increases the number of cells to be delivered but attenuates the ability of these cells to improve post-infarct function.

### Physiologic serum-free cell culture enhances paracrine production by EDCs

**Fig. 5:**
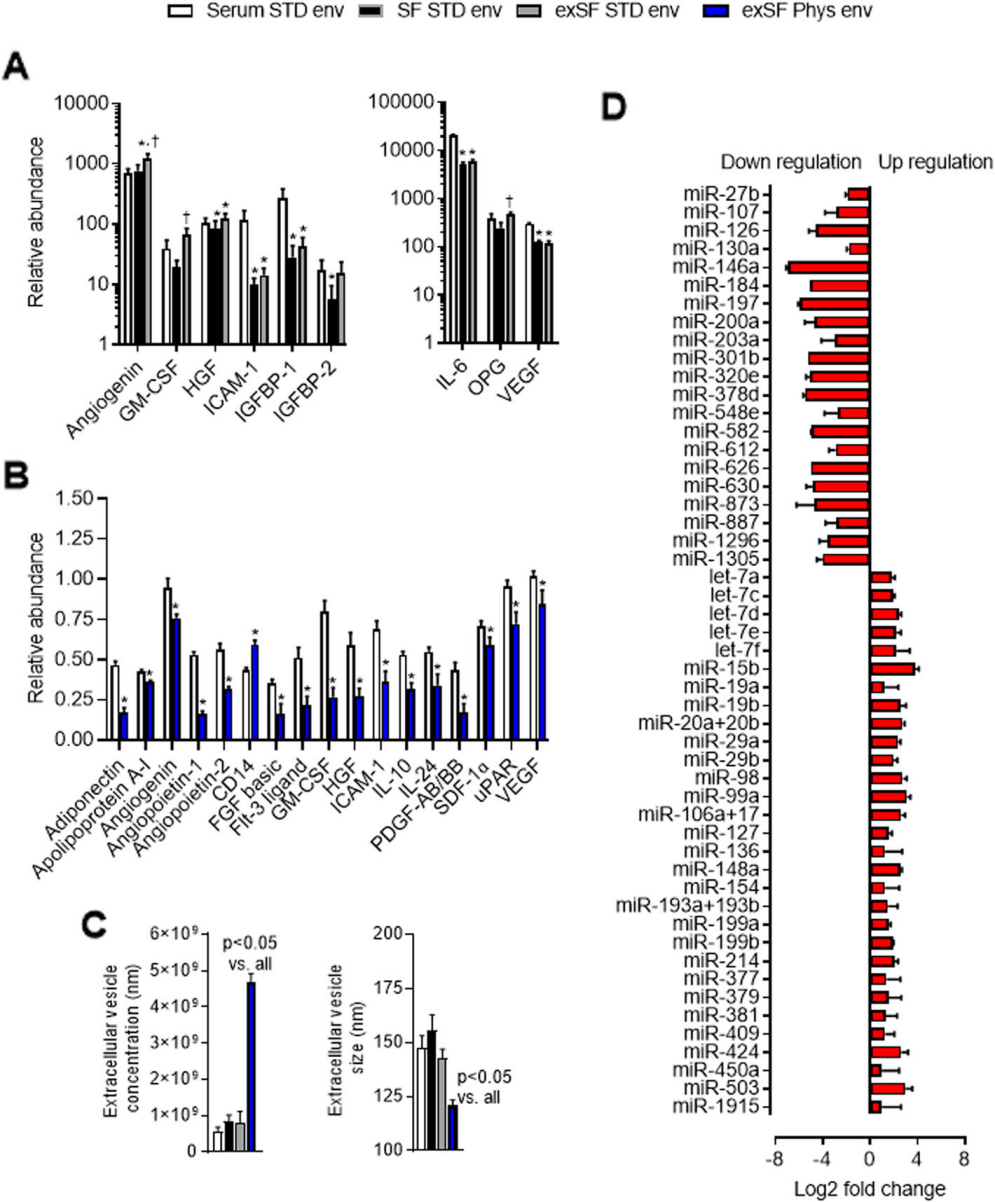
Influence of serum free culture conditions on the paracrine signature of explant-derived cells (EDCs). **a** Select cytokines from an unbiased protein array profiling cytokine content within EDC conditioned media under hypoxic (1% oxygen) basal media conditions. *p < 0.05 vs. media conditioned by EDCs cultured within standard (STD) cell conditions. *n*=4 per group. **b** Select cytokines from an unbiased proteome array showing differential expression within media conditioned by STD EDCs and exSF Phys Env EDCs. *p < 0.05 vs. media conditioned by EDCs cultured within standard (STD) cell conditions. *n*=4 per group. **c** Effect of cell culture conditions on extracellular vesicle concentration and size. *p < 0.05 vs. all. *N*=3-4 per group. **d** Up and downregulated miRNAs in EVs isolated from exSF Phys Env EDCs compared to serum STD EDCs. *n*=3. FGF basic: Fibroblast growth factor basic; Flt-3: tyrosine kinase 3; GM-CSF: Granulocyte-macrophage colony-stimulating factor; HGF: Hepatocyte growth factor; ICAM: Intercellular adhesion molecule; IGFBP: Insulin-like growth factor binding protein; IL: interleukin; OPG: Osteoprotegerin; PDGF: Platelet-derived growth factor; SDF-1α: stromal cell-derived factor 1 alpha; uPAR: urokinase-type plasminogen activator; VEGF: Vascular endothelial growth hormone

As shown in Fig. 5a and Additional file 1: Figure S4, widespread unbiased cytokine profiling of conditioned media demonstrated that transitioning cell to SF culture had minor effects on cytokine production that generally favored cells cultured under high serum conditions (*χ*^2^ value 2.7; *p* = 0.01 versus the expected frequency of cytokines elevated in serum EDC conditioned media). In contrast, transitioning cells to the controlled physiological environment enhanced the ability of these cells to produce pro-healing, anti-inflammatory, cytokines (Fig. 5b and Additional file 1: Figure S5).

Given clear evidence suggesting that a portion of cardiac-derived cell regenerative potency is dependent upon the secretion of transplanted cell-derived extracellular vesicles [31], the microparticle content within EDC conditioned media was profiled. Nanocyte tracking analysis revealed that SF culture conditions alone and SF expansion alone had no effect on microparticle production or size (Fig. 5c). In contrast, physiological conditions markedly increased extracellular vesicle production by ~ 6-fold compared to serum or SF STD conditions. Interestingly, a consistent physiological environment also modified extracellular vesicle production to sizes (120 ± 3 vs. 148 ± 4 NM, *p* < 0.05) that more closely align with accepted definitions for exosomes [32]. Despite no marked differences in treatment outcomes, the miRNA cargo within SF Phys Env EDCs as physiologic cell culture altered the expression of 51 miRNAs within EDC EVs (*p* < 0.05, Fig. 5d) while enriching exosomes with miRNA in pathways related to proliferation, myogenesis, and reduced inflammation (Additional file 1: Figure S6). Among this altered payload were miRNAs associated with cardiomyocyte proliferation (miR199 [33, 34]) or salvage (miR19a [35, 36]) and attenuation of adverse cardiac remodeling (miR148a [37]), fibrosis (miR29b [38]), and hypertrophy (miR99a [39]) which provides intriguing targets for future study.

## Discussion

Although heart-derived cell therapy has progressed rapidly from bench to bedside over the past decade [4–7], straightforward clinical translation will be hampered by reliance on traditional culture conditions which often include ill-defined or xenobiotic components. Overcoming these barriers represents a critical next step in the translation of cell therapies for clinical use. In this study, cultures of the outgrowth from plated cardiac tissue (EDCs) were undertaken to investigate the effects of a serum-free, xeno-free culture system on product identity. EDCs were chosen as the cell type to study as they represent the initial cell product used prior to antigenic selection [40] and/or prolonged inductive culture [41]. Previous work has shown that EDCs provide a complementary collection of cell types that provide degrees of myocardial repair equivalent to CDCs while retaining a 1000-fold greater capacity to adopt a cardiac fate [9], making EDCs a valuable tool to detect the early effects of divergent cell culture practices [14].

The cell culture outcomes outlined above suggest that serum-free conditions yield a cell product morphologically similar to standard cardiac explant conditions. Interestingly, the smaller and more homogeneous cell product derived using SF xenogen-free conditions likely results from stable consistent recombinant cytokines found in the defined media while retaining antigenic identities at levels consistent with traditional cultures. Notably, the use of recombinant serum-free media also avoids exposing human cells to bovine-sourced extracellular vesicles, a component of traditional media that remains poorly defined.

The media formulation chosen for this work was based our pre-clinical work comparing different SF media formulations for allogeneic bone marrow-derived mesenchymal stromal cell culture (Cellular Immunotherapy for Septic Shock: A Phase I Trial (CISS), NCT02421484). These data suggested that Nutristem XF media provided consistent culture outcomes—rationalizing its use for the CISS trial. While extension of other SF media formulations towards EDC culture is uncertain, future studies are needed to understand if important differences exist between current commercial products. Previous work by our group has identified that EDCs express important receptors (such as insulin-like growth factor 1 and SDF-1α) that may influence proliferation [21, 23]. It follows that supplementation of commercial SF media formulations may be used in the future to formulate media tailored to EDC culture outcomes.

Interestingly, eliminating ill-defined bovine-sourced cytokines or extracellular vesicles had marked effects on the cytokine profile of EDCs, suggesting that serum-free recombinant cytokine conditions permit cells to retain a more “human” or “natural” identity. This effect very likely altered the EDC-recipient interactions as we noted reduced production of key cytokines implicated in post-infarct repair (such as IL-6) which largely reflected a downshift in inflammatory cytokines [22, 30]. Effects on immune-mediated fibrosis and arteriogenesis remain to be defined [42], but as shown above, delivery of a more homogenous cell product provided greater healing within scarred tissue and superior functional gains.

**Fig. 6:**
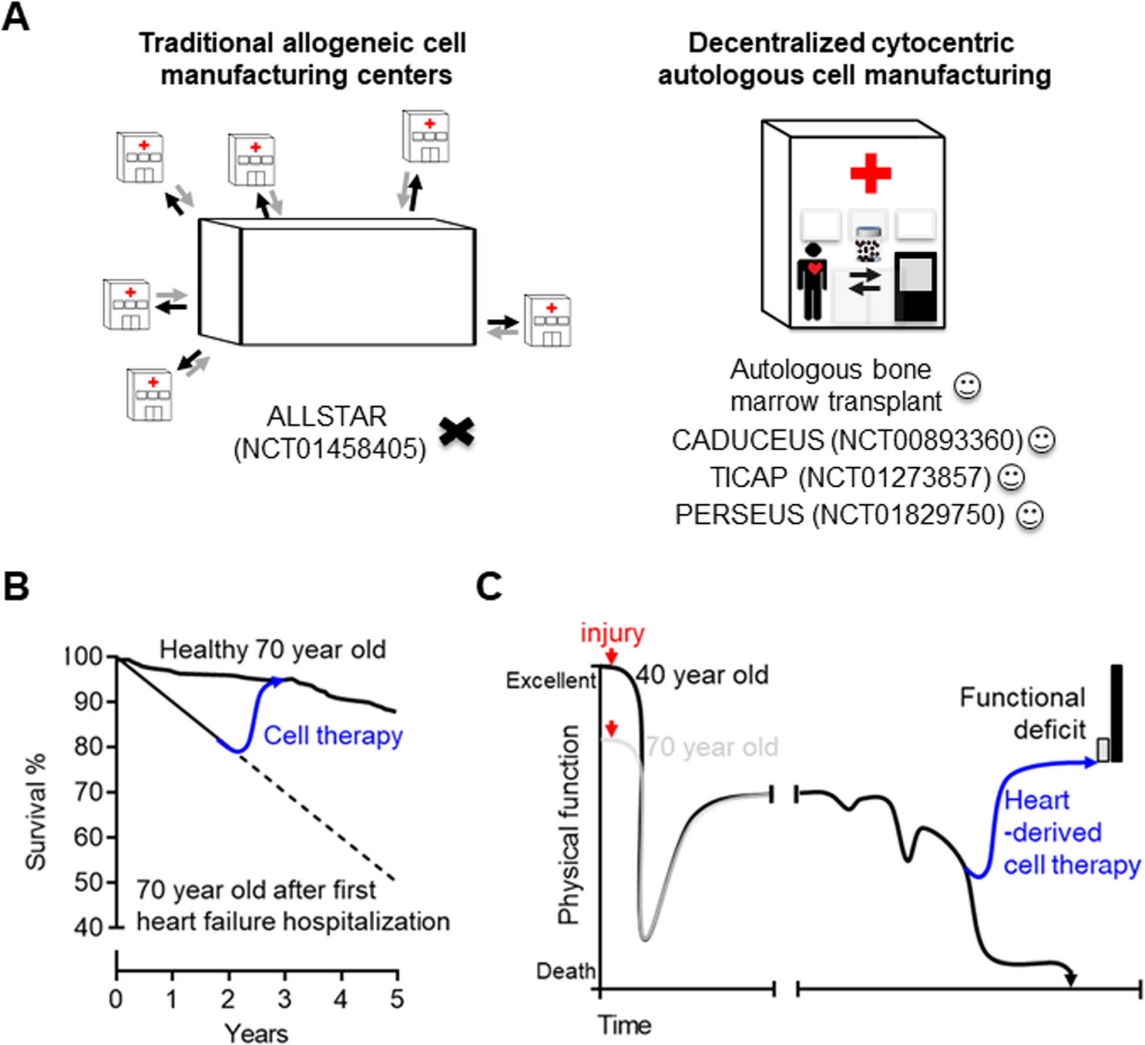
The rationale for autologous heart-derived cell therapy. **a** Schematic comparison of traditional centralized cell manufacturing with the decentralized cytocentric strategy afforded through modular physiologic GMP serum-free cell culture. **b** Conceptual promise of all cell therapies (blue line) to restore function and prolong survival contrasted with the dismal prognosis conferred after first hospitalization for heart failure. **c** Schematic outline of the real world effect of heart failure on physical function ranging between excellent and death (ordinate) as a function of time (abscissa) from cardiac injury. The influence of early medical therapy and progressive functional decline is contrasted between young (black line) and aged (gray line) patients. The ability of autologous heart-derived cell therapy (blue line) to partially restore function is shown which results in a functional deficit that for an elderly patient is not very much different from baseline. Figure modified from Goodlin with permission [43]

Transitioning serum-free cell culture to the closed Phys Env enhanced proliferation and made it possible to attain clinically relevant cell doses (i.e., 100 M cells) in 3 weeks. Continuous physiologic culture also broadened the paracrine signature of cells to amplify pro-survival signaling and treatment outcomes. While traditional open process systems (i.e., standard conditions) are necessary for early phase development, moving to closed systems eliminates the need for large costly cell manufacturing facilities. Yet current bioreactor technology is inflexible and often requires open steps in cell processing. Cytocentric isolators (such as the BioSpherix Xvivo Isolators used in this study) represent a staged approach to closed cell processing that, once the tissue enters the interconnected modules, ensures aseptic manipulation throughout the manufacturing process. This innovation permits a scale out design that could be readily employed at almost all major hospital facilities (Fig. 6a). Similar to hematopoietic bone marrow transplants, which have been provided as a successful hospital-based treatment since the 1980s around the world, product release criteria would comprise measures of identity, potency, sterility, and viability that are readily available at any major cardiac center. Decentralization would also reduce costs and logistical barriers to autologous (self to self) administration. Intriguingly, this approach finds traction within the heart-derived cell therapy literature as only autologous, decentralized, cell manufacturing trials have demonstrated to date any treatment efficacy [4–7] while traditional, centralized cell manufacturing of allogeneic therapies have failed to meet expectations [44].

With the astounding number of recent heart stem cell retractions, many have legitimately begun to question what contribution heart-derived (paracrine) therapeutics may ever provide to patient care [8]. To this, a reassuringly large body of work validates the therapeutic use of heart-derived cells but the future role of these cells is uncertain [45]. As shown in Fig. 6b, heart failure is a highly malignant disease that, despite decades of research, still kills half of all patients within 5 years of diagnosis [1]. The promise of heart-derived cell therapy to reduce this risk may not be utter hyperbole as pre-clinical data from more than 45 independent labs clearly support efficacy and clinical delivery to 100+ patients worldwide has been without any signal of harm and promising hints of efficacy (for autologous cell products). This safety record contrasts with pluripotent stem cell sources that completely restore heart function by transplanting new myocytes at the cost of life-threatening heart rhythms [46]. It also has become clear in recent years that complete cardiac restoration may not be mandatory for a therapy to be deemed clinically important. The medical literature supported this concept early on with 5–6% increases in ejection fraction improving both survival and symptomatic benefits [47–54]. Device therapy confirmed these effects with 8% increases in ejection fraction seen after electrical resynchronization translating into marked lasting improvements in patient symptoms [55–58]. Heart-derived cell therapy may suffice to partially reverse or stabilized the inexorable decline in patient function (Fig. 6c). The balance between costs and “functional deficit” will dictate the future of heart-derived cells or other paracrine therapies. We anticipate an autologous heart-derived cell product will provide a very acceptable clinical outcome for older patients whereas younger more active patients would be more motivated to pursue, and accept the risks of, pluripotent cell sources.

The study has several limitations that include the transplant patients to enable the comparison between effects of media on atrial and ventricular tissue. Patients donating ventricular biopsies were inevitable considerably healthier and younger than appendage donors, an observation reflecting their candidacy to undergo heart transplant. While these limited medical comorbidities may have inflated measures of cell proliferation, the data supports the notion that transitioning to a serum-free media formulate has limited effects on proliferation of cells from ventricular biopsies which have been used in cell therapy trials [4]. Previous work has shown that the ion channel expression controls membrane potential and proliferation of heart-derived cells [59]. While the impact of altering cell culture conditions on electrophysiologic function was not explored, this constitutes an interesting avenue for future exploration. In deference to clinical translation, a xenogenic transplant model (human cells into immunodeficient mice) was chosen for the study, and while this humanized mouse model provides a strong signal of benefit, confirmatory studies using immune-competent models need to be performed. We also observed expanded physiologic cultured cells provided intriguing shifts in the paracrine output towards a more anti-inflammatory profile; the effects of which deserve to be studied in future work.

## Conclusions

Transition of heart-derived cells to serum-free conditions provided a superior cell product without exposure to ill-defined or xenobiotic components. Implementation of continuous physiological culture provided further increases in cell growth and paracrine output which translated to increased functional benefit after experimental myocardial infarction.

## Supplementary information

**Additional file 1: Table S1.** Antigen name, function and lineage association used to profile EDCs. **Figure S1.** Influence of transitioning to SF GMP grade culture conditions on cell yields and phenotypic make-up. **Figure S2.** Influence of cell culture conditions on cardiogenic differentiation. **Figure S3.** Influence of cell culture conditions on peri-infarct vascularization. **Figure S4.** Influence of serum free (SF) standard environment (STD env) cell culture conditions on cytokine production using unbiased proteomic profiling within EDC conditioned media. **Figure S5.** Influence of EDC expansion within a physiologic environment (exSF phys env) compared to standard environment (STD env) cell culture conditions on cytokine production using unbiased proteomic profiling. **Figure S6.** KEGG pathway analysis.

## Data Availability

All data generated or analyzed during this study are included in this published article and its supplementary information files.

## Abbreviations

abcg2: ATP-binding cassette sub-family G member 2
ACEI: Angiotensin-converting enzyme inhibitors
ARB: Angiotensin II receptor blockers
α-SMA: Alpha smooth muscle actin
β-Gal: Beta-galactosidase
BMI: Body mass index
CAC: Circulating angiogenic cells
CCS: Canadian Cardiovascular Society
cTnT: Cardiac troponin T
DAPI: 4',6-Diamidino-2-phenylindole
DDR2: Discoidin domain receptor 2
EDC: Explant-derived cell
FGF basic: Fibroblast growth factor basic
GM-CSF: Granulocyte-macrophage colony-stimulating factor
GMP: Good manufacturing practices
HGF: Hepatocyte growth factor
HNA: Human nuclear antigen
HUVEC: Human umbilical vein endothelial cells
ICAM: Intercellular adhesion molecule
IGFBP: Insulin-like growth factor binding protein
IL: Interleukin
LCA: Left coronary artery
miRNA: MicroRNA
NOD-SCID: Non-obese diabetic severe combined immunodeficient
NYHA: New York Heart Association
OPG: Osteoprotegerin
PDGFR: Platelet-derived growth factor receptor
Phys: Biospherix continuous atmospheric, relative humidity, and temperature-controlled cell culture conditions
qPCR: Quantitative polymerase chain reaction
RH: Relative humidity
SDF-1α: Stromal cell-derived factor 1 alpha
SF: Serum free
SSEA-1: Stage-specific embryonic antigen-1
STD env: Standalone incubators in clean room biosafety cabinet environment
uPAR: Urokinase-type plasminogen activator
VEGF: Vascular endothelial growth factor
vWF: von Willebrand factor
