# Supplementary material for: Physiologic expansion of human heart-derived cells enhances therapeutic repair of injured myocardium
Source: Stem Cell Res Ther. 2019 Nov 4;10:316. doi: 10.1186/s13287-019-1418-3 (PMC6829847; doi:10.1186/s13287-019-1418-3)
Supplement: Supplementary file 3 — Additional file 1: Table S1. Antigen name, function and lineage association used to profile EDCs. Figure S1. Influence of transitioning to SF GMP grade culture conditions on cell yields and phenotypic make-up. Figure S2. Influence of cell culture conditions on cardiogenic differentiation. Figure S3. Influence of cell culture conditions on peri-infarct vascularization. Figure S4. Influence of serum free (SF) standard environment (STD env) cell culture conditions on cytokine production using unbiased proteomic profiling within EDC conditioned media. Figure S5. Influence of EDC expansion within a physiologic environment (exSF phys env) compared to standard environment (STD env) cell culture conditions on cytokine production using unbiased proteomic profiling. Figure S6. KEGG pathway analysis. [file 13287_2019_1418_MOESM1_ESM.docx]

**Additional Files**


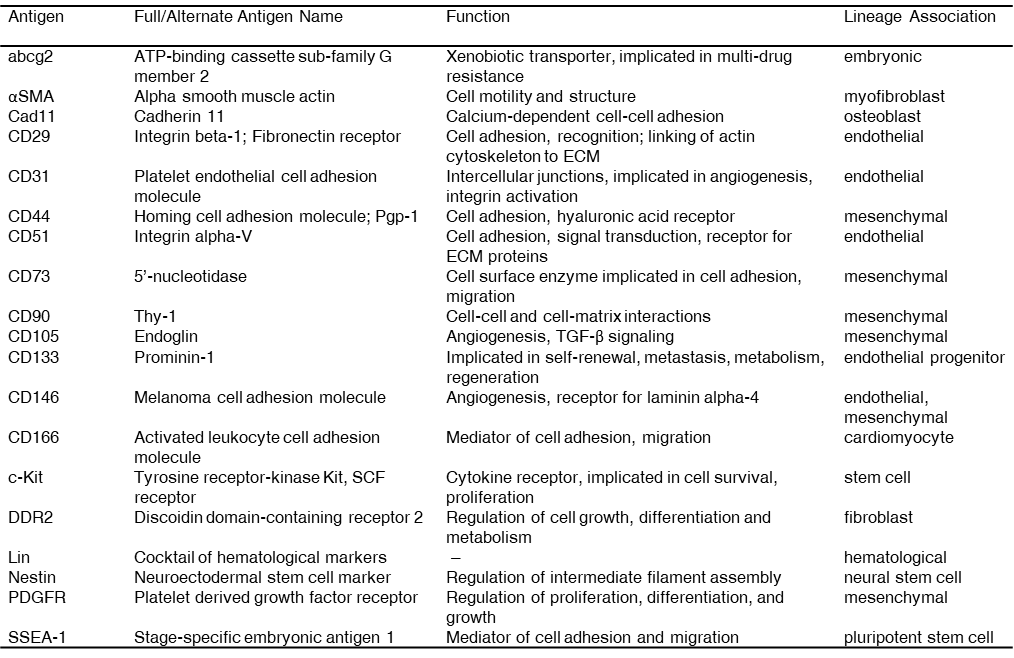


**Table S1.** Antigen name, function and lineage association used to profile EDCs.


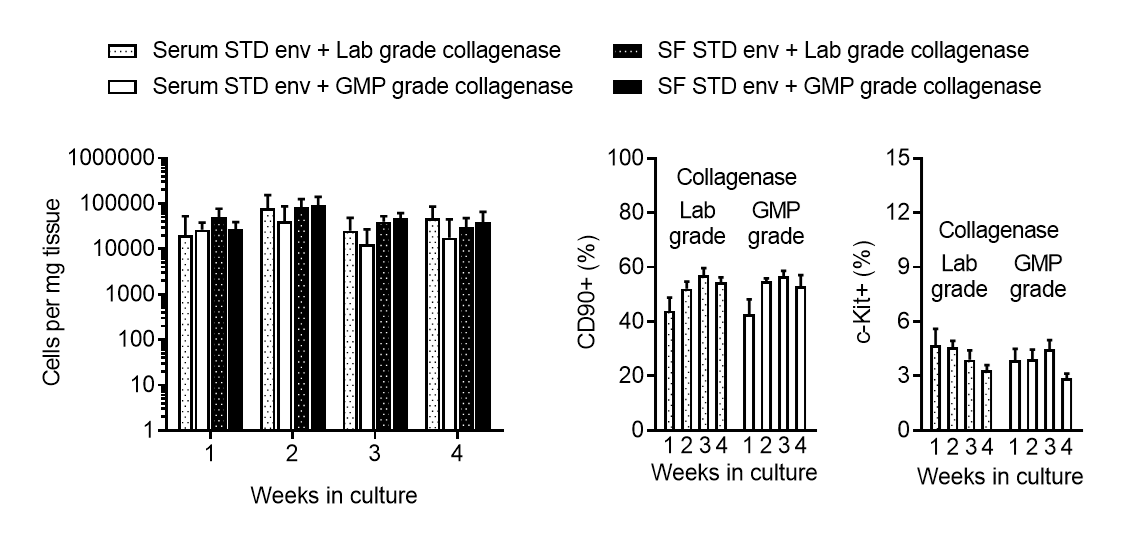


**Fig S1.**  Influence of transitioning to SF GMP grade culture conditions on cell yields and phenotypic make-up. Impact of the initial enzyme used for tissue digestion and subsequent media formulation on cell yields (left panel) and flow cytometric identity (right panel) of EDCs during serial (weekly) harvests using mild enzymatic liberation from the cultureware.

**
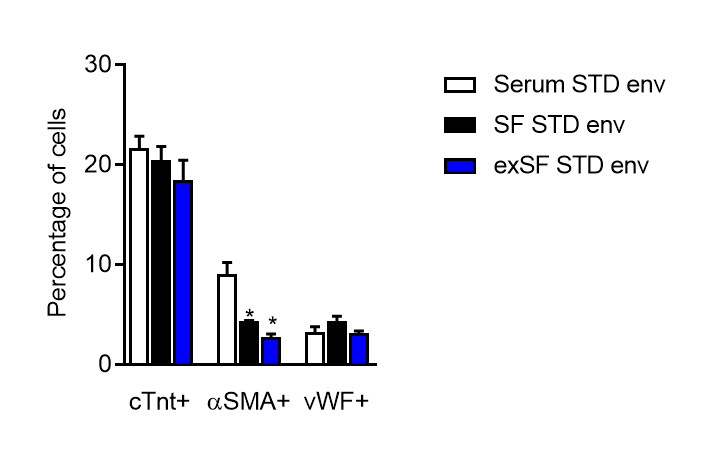
**

**Fig S2.** Influence of cell culture conditions on cardiogenic differentiation. Flow cytometric composition of cells within EDCs that expressed alpha smooth muscle actin (αSMA), cardiac troponin T (cTNT) or von Willebrand Factor (vWF) after 1 week in culture. n:4 per group. *p<0.05 versus serum STD culture.


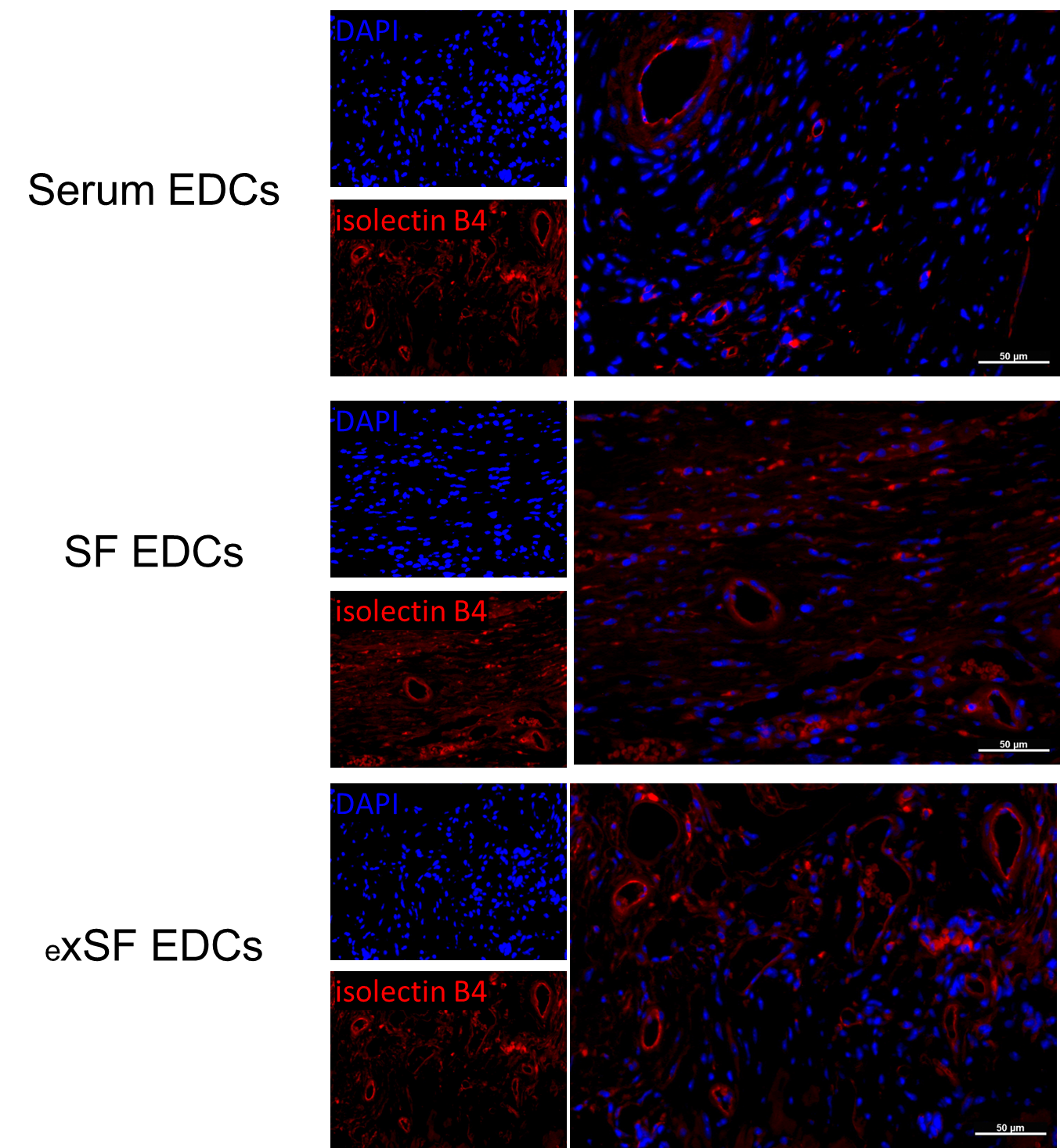


**Fig S3.** Influence of cell culture conditions on peri-infarct vascularization. Representative images demonstrating peri-infarct vascularization (isolectin B4+ cells). Scale bar: 50 μm.


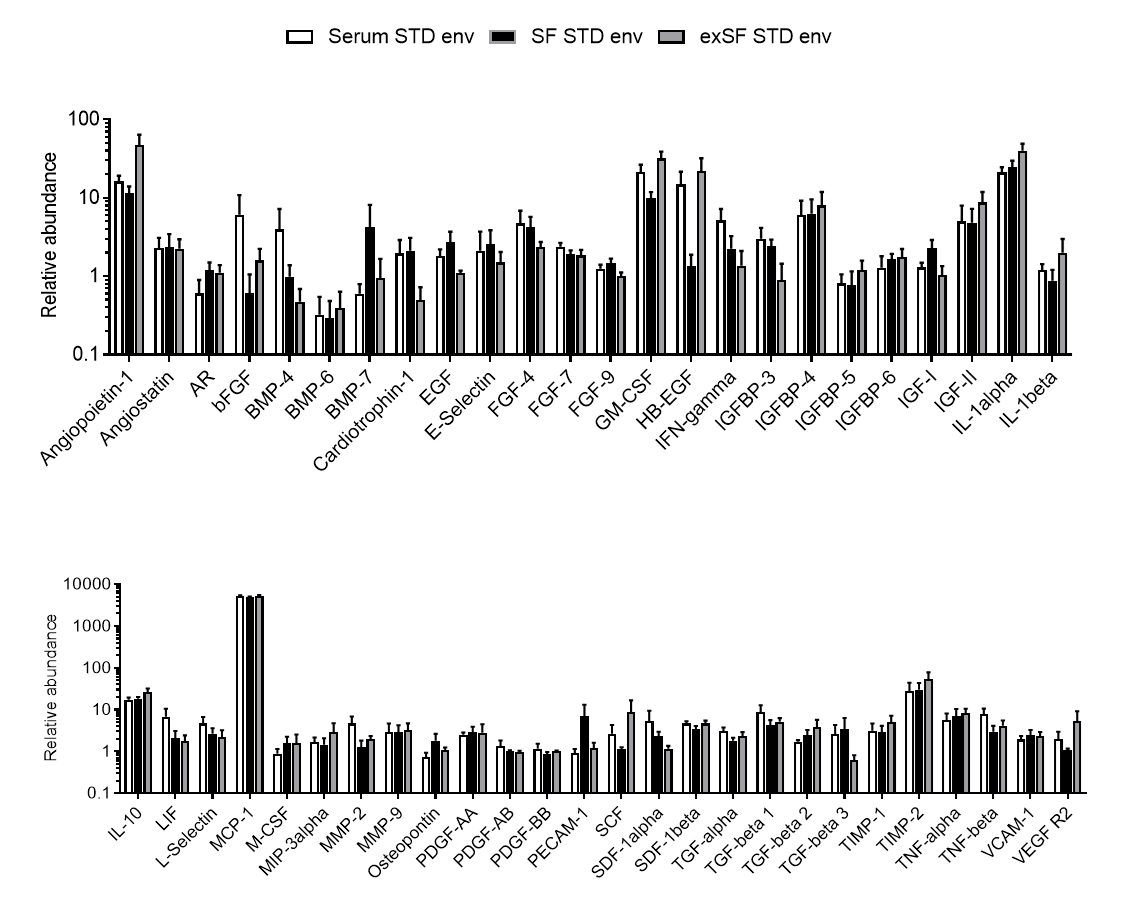


**Fig S4.** Influence of serum free (SF) standard environment (STD env) cell culture conditions on cytokine production using unbiased proteomic profiling within EDC conditioned media. n:4 per group. AR: angiotensin receptor, bFGF: basic fibroblast growth factor, BMP: bone morphogenetic protein, EGF: epidermal growth factor, FGF: fibroblast growth factor, GM-CSF: granulocyte-macrophage colony-stimulating factor, HB-EGF: heparin-binding epidermal growth factor-like growth factor, INF-gamma: interferon-gamma, IGFBP: insulin-like growth factor-binding protein, IGF: insulin-like growth factor, IL: interleukin, LIF: leukocyte inhibitory factor, MCP: monocyte chemoattractant protein, M-CSF: macrophage colony-stimulating factor, MMP: matrix metalloproteinase, PDGF: platelet-derived growth factor, PECAM: platelet endothelial cell adhesion molecule, SCF: stem cell factor, SDF: stromal cell-derived factor, TGF: transforming growth factor, TIMP: tissue inhibitors of metalloproteinases, TNF: tumor necrosis factor, VCAM: vascular cell adhesion
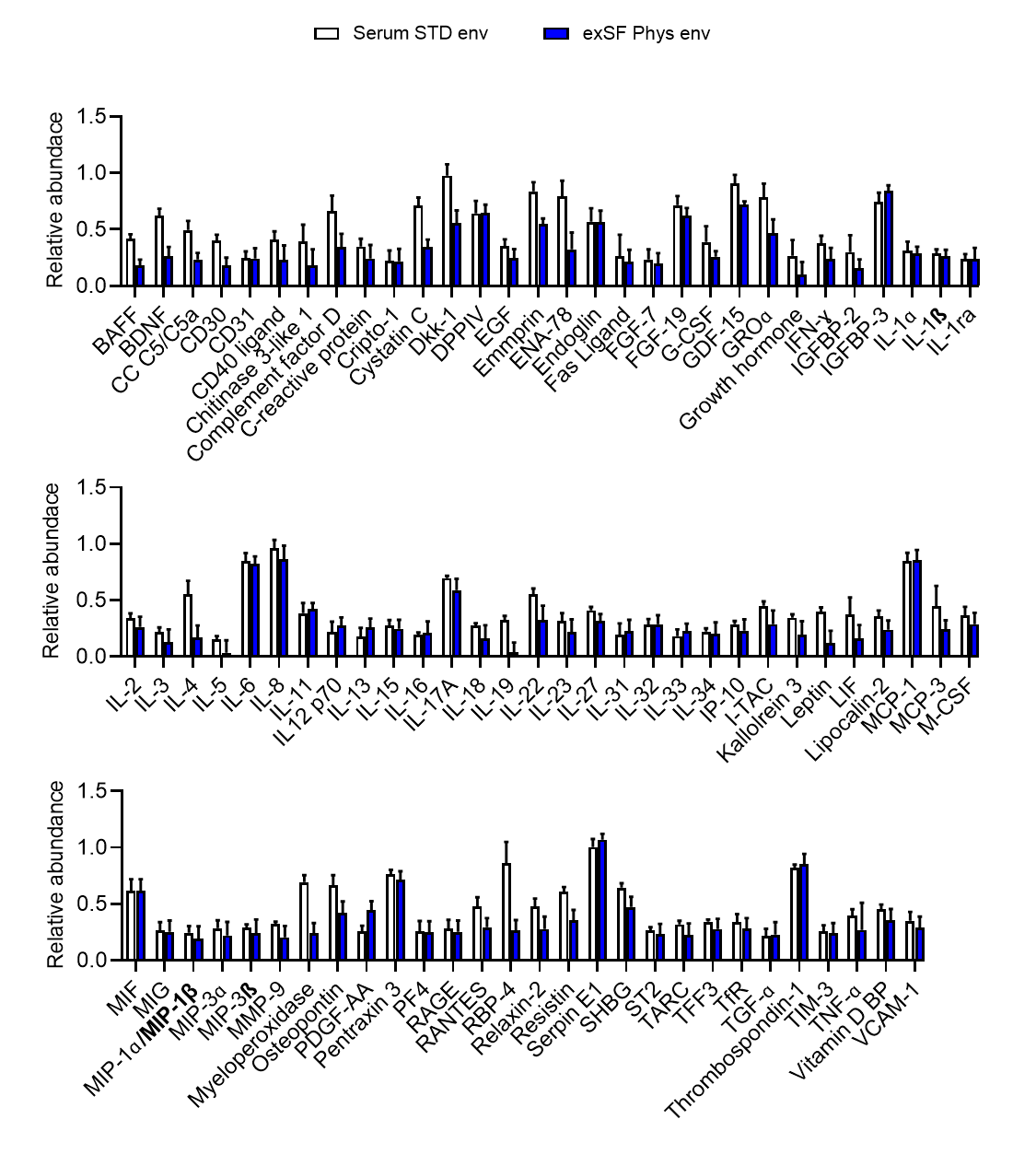
molecule, VEGF: vascular endothelial growth factor.

**Fig S5.**  Influence of EDC expansion within a physiologic environment (exSF phys env) compared to standard environment (STD env) cell culture conditions on cytokine production using unbiased proteomic profiling. n:3 per group. BAFF: B-cell activating factor, BDNF: brain-derived neurotrophic factor, CC: complement component, CD: cluster of differentiation, Dkk: Dickkopf-related protein, EGF: epidermal growth factor, ENA: epithelial-derived neutrophil-activating peptide, FGF: fibroblast growth factor, G-CSF: granulocyte-colony stimulating factor, GDF: growth differentiation factor, GRO: growth-regulated oncogene, IFN-γ: interferon gamma, IGFBP: insulin-like growth factor-binding protein, IL: interleukin, IL-1RA: interleukin-1 receptor antagonist, IP: interferon gamma-induced protein, I-TAC: interferon-inducible T-cell alpha chemoattractant, LIF: leukemia inhibitory factor, MCP: monocyte chemoattractant protein, M-CSF: macrophage colony-stimulating factor, MIF: macrophage migration inhibitory factor, MIG: monokine induced by gamma interferon, MIP: macrophage inflammatory protein, MMP: matrix metalloproteinase, PDGF: platelet-derived growth factor, PF: platelet factor, RAGE: receptor for advanced glycation endproducts, RBP: retinol binding protein, SHBG: sex hormone binding globulin, ST: suppression of tumorigenicity, TARC: thymus and activation-regulated chemokine, TFF: Trefoil factor, TfR: transferrin receptor, TGF: Transforming growth factor, TIM: T-cell immunoglobulin and mucin-domain containing, TNF: tumor necrosis factor, Vitamin D BP: vitamin D binding protein, VCAM: vascular cell adhesion molecule.

**Fig S6.** KEGG pathway analysis. Pathways associated with down- or up-regulated miRNAs in EVs isolated from SF Phys Env EDCs compared to serum STD Env EDCs (mirPath v.3 using DIANA Tools).
